# Violence and Restrictive Practices Reduced by Hospital Relocation

**DOI:** 10.1002/brb3.70760

**Published:** 2025-09-08

**Authors:** Louise Karstoft Beck, Morten Deleuran Terkildsen, Harry Kennedy, Christian Jentz, Astrid Jacobsen Harpøth, Anders Helles Carlsen, Lisbeth Uhrskov Sørensen

**Affiliations:** ^1^ Department of Forensic Psychiatry Aarhus University Hospital Psychiatry Aarhus Denmark; ^2^ Psychiatric Hospital Horsens Denmark; ^3^ DEFACTUM, Public Health Research Central Denmark Region Aarhus Denmark; ^4^ Department of Clinical Medicine, Health Aarhus University Aarhus Denmark; ^5^ Trinity College University of Dublin Dublin Ireland; ^6^ Steno Diabetes Center Aarhus Aarhus University Hospital Aarhus Denmark; ^7^ Sapienza University of Rome Rome Italy

**Keywords:** architecture, aggression, coercion, psychiatry, restrictive practices, violence

## Abstract

**Background:**

Ward design is increasingly recognized as influencing the treatment of psychiatric inpatients. However, evidence on how improved structural surroundings affect aggression and restrictive practices in forensic psychiatry is limited. To our knowledge, no studies have focused on the effect of improved treatment facilities on aggressive behavior and the prescription of restrictive practices among forensic psychiatric inpatients.

**Aim:**

This study aims to explore whether improved architectural design reduces aggression and prescription of restrictive practices in a population of forensic psychiatric inpatients.

**Methods:**

This retrospective, observational, and longitudinal study included a follow‐up 2 years prior and 2 years post relocating the Department of Forensic Psychiatry at Aarhus University Hospital Psychiatry (DFP‐AUHP) from an old hospital building to new and purpose‐built psychiatric facilities. We included all patients admitted to DFP‐AUHP during the study period; 230 unique patients before and 196 unique patients after the relocation. We compared aggression as measured by the Brøset Violence Checklist (BVC) and prescription of restrictive practices prior to and after the intervention.

Data were drawn from the Business Intelligence data portal, which routinely stores data from the Electronic Patient File. Statistical analyses were conducted to explore patient demographics, aggression, and the prescription of restrictive practices.

**Results:**

Overall, restrictive practices were more than halved among the included population of forensic psychiatric patients after the relocation (548 to 246, *p* value: < 0.001). Additionally, the total daily mean BVC, an indicator of aggressive behavior, was significantly lower post‐relocation (from 0.40 (95% CI: 0.39–0.42) to 0.27 (95% CI: 0.26–0.28), *p* value: < 0.001). Both summative data and underlying time trends evidence the results.

**Conclusion:**

Our study suggests a significant reduction in aggressive behavior and restrictive practices following the relocation. The study contributes to understanding how environmental changes can relate to patient outcomes and may be relevant for the design and renovation of psychiatric hospital facilities.

**Trial Registration:**

Central Denmark Region: 1‐16‐02‐137‐24

## Graphical abstract

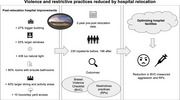

## 1 Introduction

Patient‐aggressive behavior is a frequent challenge in psychiatric facilities, causing physical and psychological harm to both patients and healthcare workers. Approximately one‐third of violent or aggressive incidents in healthcare settings result in injuries to staff members, which emphasizes the severity of this complex issue (Bowers et al. 2011).

Patient's aggressive behavior and the associated risk of violence can be reliably predicted by the Brøset Violence Checklist (BVC), which assesses the presence or absence of six key behaviors: confusion, irritability, boisterousness, verbal threats, physical threats, and attacks on objects (Hvidhjelm et al. 2014). Measuring aggressive behavior in psychiatric care can reduce violence and, consequently, restrictive practices (Hvidhjelm et al. 2023; Kennedy et al. 2020; Beghi et al. 2013). The prescription of restrictive practices to prevent or treat aggressive or violent behavior among psychiatric patients is considered a last resort due to its far‐reaching negative consequences for both the patients subjected to the intervention and the staff implementing it (Tingleff et al. 2017).

A therapeutic and safe environment with less patient‐aggressive behavior may be influenced by procedural, relational and structural factors (Seppänen et al. 2018). Procedural factors include policies for systematic risk assessment and risk management, whereas the relational factors encompass the ratio of staff to patient at ward level and the therapeutic relationship between inpatients and healthcare staff (Allen 2023). The structural conditions encompass the design and the architecture of the hospital (Kennedy 2002; The‐Quality‐Network‐for‐Forensic‐Mental‐Health‐Services 2021).

Reviews suggest that the architectural design of the ward plays a role in reducing restrictive practices (Oostermeijer et al. 2021) and that architecture not only fulfils basic hygienic and security requirements but also contributes to patient recovery (Bodryzlova et al. 2024).

Architectural design features that impact restrictive practices are identified in individual studies (Ulrich et al. 2018; van der Schaaf et al. 2013; Czernin et al. 2024; Rohe et al. 2017; Harpøth et al. 2022). Key design elements across these studies include enhanced spatial openness and visibility, improved access to natural light and therapeutic outdoor environments, patient‐centered interior design, integration of activity spaces, and facilitation of patient‐staff interaction.

Although increasing evidence suggests that architectural design influences the use of restrictive practices in psychiatric care, systematic reviews (Grynevych et al. 2024; Papoulias et al. 2014) also highlight the need for further research before a definitive causal relationship can be established.

In 2018, the Department of Forensic Psychiatry at Aarhus University Hospital Psychiatry (DFP‐AUHP) moved from old buildings to modern, purpose‐built treatment facilities as part of a new, large university hospital integrating psychiatric and somatic healthcare facilities. One of the primary objectives was to strengthen patient, staff, and community safety. The relocation created a unique opportunity to explore how safer and updated treatment facilities influence the behavior of forensic psychiatric inpatients.

To our knowledge, no prior studies have systematically examined the impact of improved treatment facilities on aggressive behavior as measured by BVC and restrictive practices in forensic psychiatric departments. Therefore, we aimed to explore if improved treatment facilities reduced aggression as measured by the BVC and the prescription of restrictive practices in a medium‐secure forensic psychiatric department.

## 2 Methods

### 2.1 Setting

Central Denmark Region (CDR) is one of five self‐contained regions in Denmark, including around 1/5 of the Danish population (Regional‐Denmark n.d.). The psychiatric hospital services in CDR are located at one university hospital in Aarhus (AUHP) and four regional hospitals around the region (Psykiatrien‐i‐Region‐Midtjylland n.d.).

### 2.2 Forensic Psychiatry in Denmark

In Denmark, a forensic psychiatric patient is an individual who has committed an offence, due to a mental disorder, and has received a psychiatric measure ruling instead of a penal sentence. The prevalent number of unique Danish forensic psychiatric patients is close to 3000 (Justitsministeriets‐Forskningskontor 2023), and there are around 400 medium‐ and high‐secure beds (Danske‐Regioner 2018).

### 2.3 Relocation

In November 2018 DFP‐AUHP relocated from old hospital buildings to new and modern treatment facilities. The vision for the new psychiatric hospital emphasized safer, evidence‐based treatment customized to individual patient preferences. Additionally, the relocation aimed to improve life expectancy for psychiatric patients through closer integration with somatic care (KPC‐Team 2014).

The new buildings feature high safety standards and updated facilities, exemplified by strategically placed staff offices for optimal ward oversight. The design shields the courtyard, eliminating the need for fences that were present in the old facility. Several anxiety‐reducing installations, such as nature‐inspired art, were also introduced. Key improvements in the new buildings include more spacious common patient areas, increased natural light, ensuite bathrooms for all patients, and extended courtyard access (Table 1). Although the physical surroundings and treatment facilities were modernized, the patients and staff remained the same as prior to the relocation.

**TABLE 1:** Architectural differences between old and new forensic psychiatric hospital buildings at the Department of Forensic Psychiatry at Aarhus University Hospital Psychiatry (DFP–AUHP).

	Pre‐relocation^a^ Old building	Post‐relocation^b^ New building
**Department**		
Hospital building, m^2^	6975	9612
Number of wards	4	4
**Wards**		
Ward, m^2^	1185	1159
Number of patients per ward	16.3	16
Number of m^2^ per patient	73	72
Hall length, m	48	81
Hall width, m	2.2	2.4
Dynamic artificial lighting^c^	0%	100%
MD office in‐ward	25%	100%
**Inpatient room**		
Room, m^2^	17	18.3
Ensuite bathroom	22%	100%
Window size, m^2^	2.5	3.7
Rooms with a view^d^	20%	100%
Natural light, lux^e^	292	730
**Interior**		
Tv in bedroom provided	50%	100%
**Common areas**		
Dining area, m^2^	50.1	86.8
Activity rooms in ward, m^2^	40.7	72.5
Tv area size, m^2^	85.4	29.9
Number of common rooms m^2^ per patient	10.8	11.8
**Outdoor spaces within ward**		
Open air yard, m^2^	1240	421
Fencing as outside perimeter	Yes	No
Open access to yard, hours	2	12
**Safety**		
Shielded rooms	1.5	2
Shielded smoking area	0	1

^a^ Pre‐relocation period is defined from November 1, 2016 to October 30, 2018.

^b^ Post‐relocation period is defined from December 1, 2018 to November 30, 2020.

^c^ Special light that filters out blue light and features automatic dimming. The light can also be adjusted by the staff.

^d^ Clear view without buildings for a minimum of 100 m.

^e^ Light was measured within an hour in similar rooms of both the old and new hospital buildings under comparable weather conditions. Measurements were taken at the center of the room and near the door (farthest from the window) using an EC1 Hagner Digital Luxmeter.

The European Committee for the Prevention of Torture visited DFP‐AUHP in 2024 and praised the excellent living conditions, particularly the secure yet non‐carceral environment (The‐European‐Committee‐for‐the‐Prevention‐of‐Torture‐and‐Inhuman‐or‐Degrading‐Treatment‐or‐Punishment‐(CPT) 2024).

DFP‐AUHP is divided into four medium‐secure wards (R1, R2, R3, R4) with 16 beds each. Bed occupancy is mandatory at 100%.

R1 is an acute ward with forensic remand patients, individuals undergoing forensic psychiatric assessment, and patients who have been sentenced but are awaiting rehabilitative services. R2 and R4 are rehabilitation wards for patients with severe mental disorders and a psychiatric measure ruling. R3 is a specialized ward exclusively for forensic psychiatric patients from Greenland, admitted under an agreement with the Greenlandic government.

### 2.4 BVC

The BVC can predict aggressive patient behavior and, thereby, the risk of violence. Forensic psychiatric inpatients are routinely scored three times daily at DFP‐AUHP. The scores are mandatory and documented in electronic journals in standardized form for each patient. Staff are trained in using the BVC through e‐learning courses, peer training, and regular brush‐up courses.

### 2.5 Restrictive Practice

Restrictive practices refer to using measures without informed consent, as defined by the Danish Mental Health Act (MHA) (Bekendtgørelse af lov om anvendelse af tvang i psykiatrien m.v. 2024). Restrictive practices are applicable only to patients admitted to a psychiatric hospital and must be prescribed by a psychiatrist. The MHA applies equally to both forensic and non‐forensic psychiatric inpatients. Forensic psychiatric patients are admitted following a court‐ordered treatment measure under the penal code.

We measured all restrictive practices, including mechanical and manual restraint, forced treatment (acute and prolonged medication, ECT, nourishment, and treatment for life‐threatening somatic conditions), and enhanced monitoring by staff.

The psychiatrist documents restrictive practices in a standardized form in the patient's electronic journals for each prescription, which is then automatically registered in the official national register for restrictive practices.

### 2.6 Study Design and Study Population

We conducted an observational, longitudinal, and retrospective pre‐ and post‐intervention study. We included admissions for all individual patients admitted to the forensic psychiatric wards at DFP‐AUHP from November 1, 2016 to November 30, 2020. November 2018 was excluded because this was the month of relocation.

Patients were included in the study cohort when admitted to DFP‐AUHP and excluded when discharged or at the end of the study period.

An outlier who had been prescribed an extreme number of restrictive practices *before the relocation* was excluded as this was not considered representative.

### 2.7 Total Dataset

The dataset included 230 unique patients before and 196 unique patients after the relocation.

### 2.8 Data and Outcome Measures

Data was extracted from the Business Intelligence (BI) portal, which collects data from the Electronic Patient Record (EPJ) in CDR.

The dataset comprises a complete sample of all included patients and corresponding outcomes at DFP‐AUHP during the study period.

The outcomes were aggression as measured by BVC and prescription of restrictive practices according to the MHA.

We included descriptive patient population data, including sex, age, and diagnosis. According to the International Classification of Diseases, the tenth revision, the highest‐ranking diagnosis for each admission was selected.

### 2.9 Statistical Analysis

Descriptive statistics were applied to quantify the characteristics of the study population, BVC, and restrictive practices. Chi‐square tests were used when examining categorical data, and student *t*‐tests were used when analyzing continuous data.

The BVC was analyzed as the mean BVC for the population in the four daily time slots: 00 a.m. to 05.59 a.m., 06 a.m. to 11.59 a.m., 12 p.m. to 17.59 p.m., and 18 p.m. to 23.59 p.m. Each patient was typically scored three times during the day. Patients contributed a maximum of one score per time slot. If a patient was scored more than once in a time slot, the highest BVC score was selected. The mean BVC was calculated as the total sum of BVC scores per time slot divided by the number of patients scored per time slot.

Negative binomial regression models with a linear spline for time, incorporating a knot at the time of relocation, were used to estimate the mean daily rate of restrictive practice. Similarly, the mean daily level of BVC was estimated using linear regression models, including a linear spline, over time. These models were compared to a model that maintained a constant linear slope throughout the study period using likelihood‐ratio tests.

The mean daily rate of restrictive practices and the mean daily level of BVC per month were calculated for visual presentation.

Data management and analysis were performed using Stata 18 software.

## 3 Results

### 3.1 Descriptive Data

The patient population comprised 230 unique patients before and 196 unique patients after the relocation, who were similar regarding gender (91% male vs. 90% male), age (mean age of 35 years vs. 35 years), and primary diagnosis (49% vs. 56% with psychotic disorders). Similarly, the other diagnostic pathways remained unchanged.

### 3.2 BVC

Data included 45.588 BVC before and 45.029 BVC after the relocation.

The overall daily mean BVC was significantly lower after the relocation compared to before; from 0.40 (95% CI: 0.39–0.42) to 0.27 (95% CI: 0.26–0.28), (*p* value: < 0.001), (Table 2).

**TABLE 2:** Total number of restrictive practices, selected categories of specified restrictive practices, and total mean of BVC at the Department of Forensic Psychiatry at Aarhus University Hospital Psychiatry (DFP‐AUHP) pre and post the relocation.

	Pre‐relocation^a^ Old hospital	Post‐relocation^b^ New hospital	*p* value
Total number of restrictive practices	548	246	< 0.001
Total number of mechanical restraints	186	68	< 0.001
Total number of involuntary acute medications	127	55	< 0.001
Overall daily mean BVC (95%, CI) Total mean BVC timeslot 00.00–05.59 a.m. (95%, CI) Total mean BVC timeslot 06.00–11.59 a.m. (95%, CI) Total mean BVC timeslot 12.00–17.59 p.m. (95%, CI) Total mean BVC timeslot 18.00–23.59 p.m. (95%, CI)	0.40 (0.39–0.42) 0.29 (0.25–0.33) 0.22 (0.20–0.25) 0.18 (0.17–0.19) 0.17 (0.17–0.18)	0.27 (0.26–0.28) 0.30 (0.25–0.35) 0.32 (0.29–0.35) 0.13 (0.12–0.13) 0.11 (0.10–0.11)	< 0.001 0.783 < 0.001 < 0.001 < 0.001

^a^ Pre‐relocation period is defined from November 1, 2016 to October 30, 2018.

^b^ Post‐relocation period is defined from December 1, 2018 to November 30, 2020.

**FIGURE 1:**
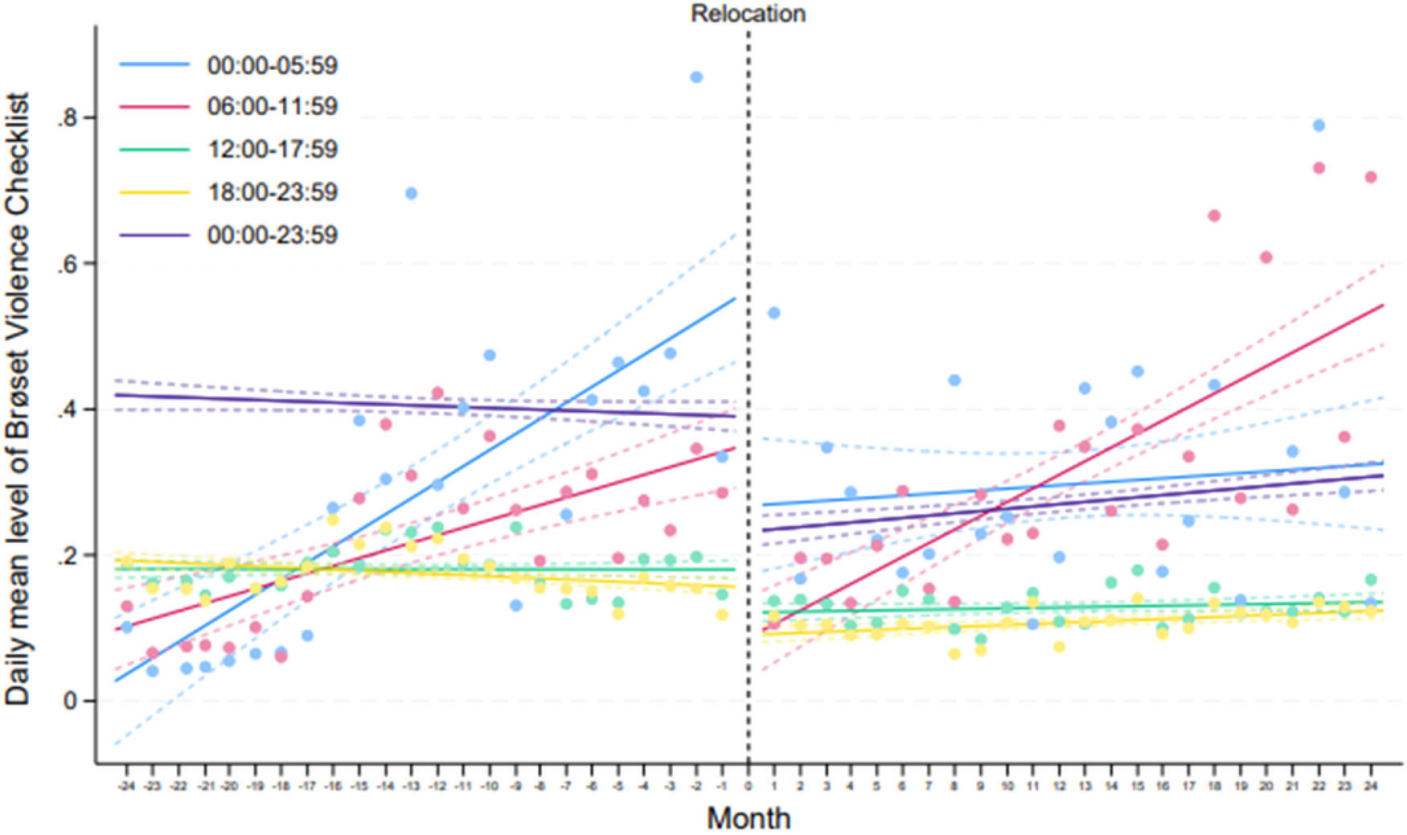
Daily mean Brøset Violence Checklist (BVC) 2 years pre‐ and 2 years post‐relocation of DFP‐AUHP.

The underlying trend for the daily mean BVC from 00.00 to 23.59 o'clock is significantly lower after the relocation than before (Figure 1).

The daily mean BVC showed a stable trend between 12:00 p.m. and 23:59 p.m. both before and after the relocation but significantly lower after the relocation. Between 06:00 a.m. and 11:59 a.m., the trend is upward before and after the relocation but with a steeper incline post‐relocation. From 00:00 a.m. to 05:59 a.m., the trend is upward before the relocation, while after the relocation, the trend becomes less pronounced and more stable (Figure 1).

### 3.3 Restrictive Practices

The total number of restrictive practices was reduced by more than half following the relocation (548 to 246, *p* value: < 0.001) (Table 2).

**FIGURE 2:**
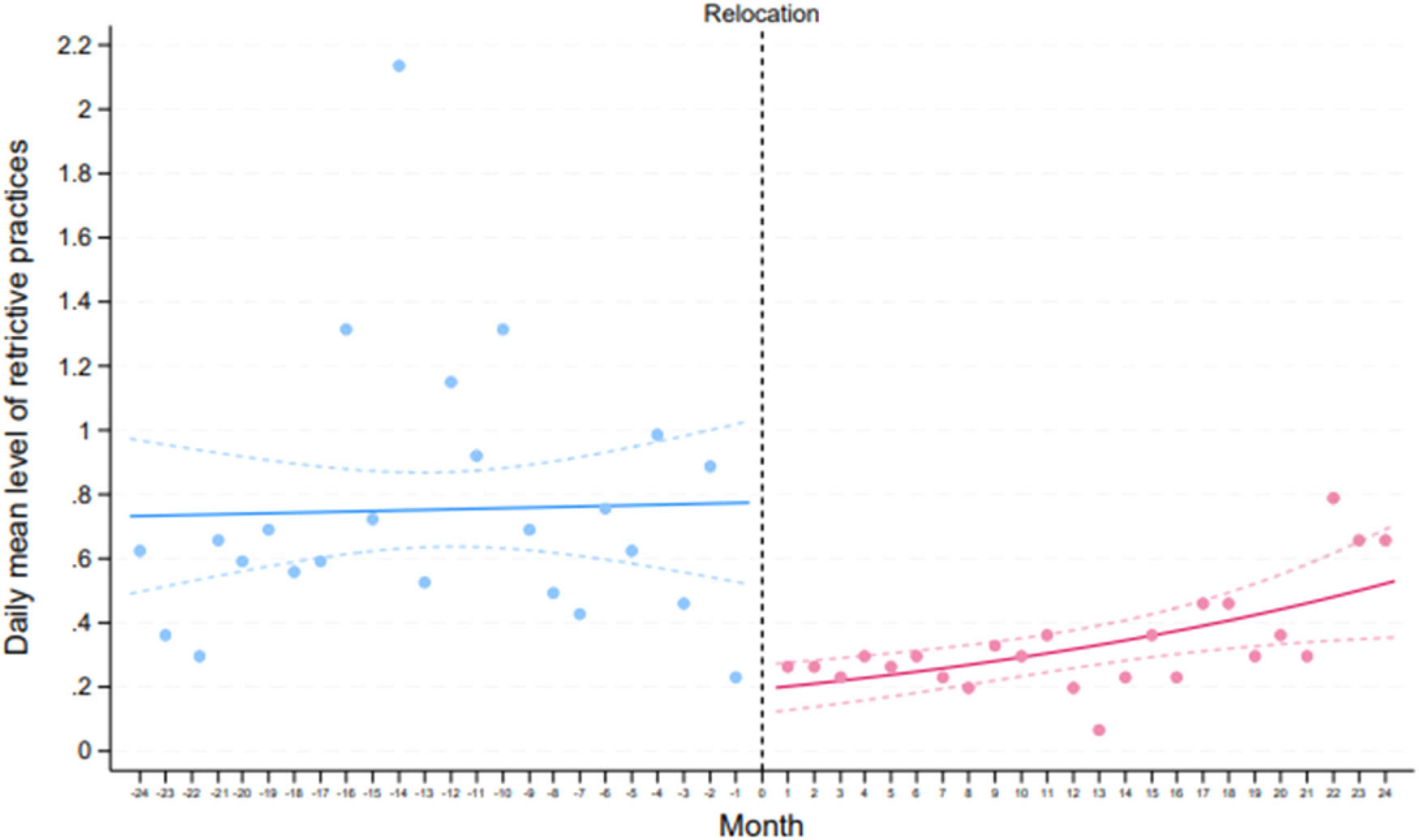
The daily mean level of restrictive practice 2 years pre‐ and 2 years post‐relocation of DFP‐AUHP.

The underlying trend for the daily mean of all restrictive practices remained stable prior to the relocation, decreased significantly, and remained lower for up to 21 months after the relocation. However, a minor upward trend was observed after the relocation, primarily driven by the last 3 months (months 22–24) (Figure 2).

**FIGURE 3:**
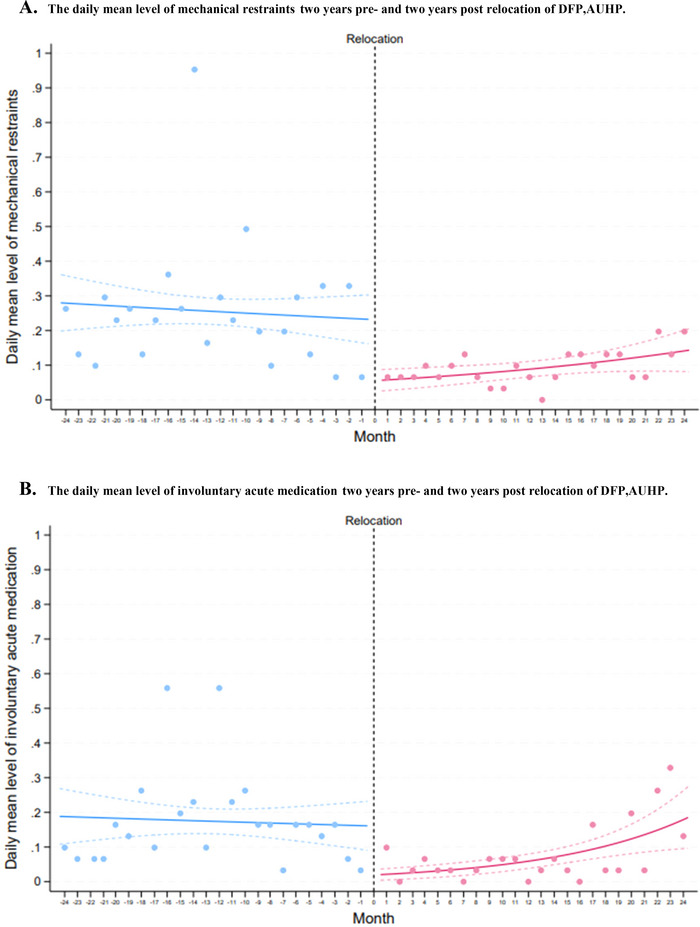
(A) The daily mean level of mechanical restraints 2 years pre‐ and 2 years post‐relocation of DFP‐AUHP. (B) The daily mean level of involuntary acute medication 2 years pre‐ and 2 years post‐relocation of DFP‐AUHP.

The total number of mechanical restraints decreased by nearly two‐thirds following the relocation (186 to 68, *p* value: < 0.001). The use of involuntary acute medication also more than halved after the relocation (127 to 55, *p* value: < 0.001), (Table 2). Thus, there was no increase in other forms of restrictive practices. The underlying trend for the daily mean of mechanical restraints was significantly lower after the relocation compared to before. However, a slight upward trend was observed for the last 3 months (months 22–24) following the relocation, primarily driven by this recent period (Figure 3A). Similarly, the underlying trend for the daily mean of acute involuntary medication administrations was statistically significantly reduced post‐relocation, although a slight upward trend in the last 3 months was also seen (Figure 3B).

## 4 Discussion

### 4.1 Summary of Main Results

We conducted a retrospective, observational, and longitudinal study with a follow‐up 2 years prior and 2 years post the relocation of DFP‐AUHP from old hospital buildings to safer, purpose‐built hospital facilities (Table 1).

Our first main finding was a significant reduction in the total number of restrictive practices, including mechanical restraints and involuntary acute medications, following the relocation compared to the period before (Table 2). This decrease was also evident when examining the underlying trend over time across all three categories (Figures 2 and 3A,B).

Our second key finding was a significant reduction in overall aggression, as measured by the BVC, following the relocation compared to the period before (Table 2). This decline was also reflected in the underlying trends over time (Figure 1).

### 4.2 Discussion of the Results and Existing Research in This Field

To our knowledge, this is the first study to examine both BVC‐measured aggression and restrictive practices in a forensic psychiatric population. The findings support growing evidence that facility design influences aggression and the prescription of restrictive measures.

Ulrich et al. (2018) identified ten stress‐reducing design elements for psychiatric wards, such as access to natural light, outdoor spaces, noise reduction elements, and spaces to maintain privacy. Our empirical study demonstrated that several of these elements were implemented in the new hospital building at DFP‐AUHP (Table 1). In our study, measured light levels more than doubled in the new facility (from 292 to 730 lux, Table 1). Similarly, access to outdoor areas increased substantially (12 h vs. 2 h, Table 1). Furthermore, the proportion of rooms with larger windows and a view increased from 20% to 100%, and patient rooms with ensuite bathroom increased from 22% to 100% (Table 1).

Ulrich et al. (2018) tested the model of ten stress‐reducing design elements in a hospital that relocated from an old building with only 1 of 10 stress‐reducing elements to a new facility featuring 9 out of 10 elements. Additionally, a control hospital with 1 out of 10 features and no relocation was included in the study. Ulrich's study reported a 50% reduction in physical restraints and a slight decline in injections after relocation to the new hospital. Similarly, our findings showed that both restrictive practices and acute involuntary medication administrations were reduced by more than half following the relocation. Both Ulrich's and our findings contribute to the growing evidence that architectural design may play a critical role in reducing restrictive practices in psychiatric care. However, as multiple factors changed simultaneously during both relocations, it remains challenging to isolate the specific elements driving the observed outcomes. Although the exact mechanisms are unclear, we argue that these combined changes collectively create a less stressful environment, which may help reduce aggression and prescription of restrictive practices. Further research is needed to clarify these relationships and to identify which environmental elements are most critical for improving patient outcomes.

Another study by Czernin et al. (2024) also examined how architectural features influence restrictive practices in child and adolescent psychiatric wards. Similar to both our study and Ulrich et al. (2018), this research examined restrictive practices before and after an architectural intervention which involved rebuilding of the department with increased ward space, a shift from two‐ and four‐bed rooms to single or double bedrooms, direct access to a garden, improved sanitary facilities and more natural light. After the relocation, significantly fewer patients were affected by restrictive practices which aligns with the findings in both Ulrich's study and ours. However, our study differs from Ulrich's and Czernin's since we focus on forensic psychiatric patients.

Several studies (Ulrich et al. 2018; Czernin et al. 2024) have examined the impact of a hospital relocation on patient behavior by analyzing the total number of restrictive practices as a total count of events over a specific period before and after the relocation and thereby compared the old and new buildings. Our study, alongside Harpøth et al. (2022), takes a more nuanced approach by using linear regression to examine trends in restrictive practices over time around the relocation. While before‐and‐after comparisons offer a simple overview, they may overlook important trends, potentially leading to inaccurate conclusions. By examining underlying trends, we provide a more nuanced understanding of long‐term changes that summative analyses might miss. Although more time‐consuming, this approach enables a more detailed evaluation of relocation effects. We encourage further research that examines trends over time rather than relying on simple summative comparisons.

### 4.3 Strengths and Limitations

Our study achieves complete case ascertainment as data is sourced from the BI‐portal, which automatically captures and stores all patient journal entries, minimizing the risk of selection bias and ensuring comprehensive coverage of the study population.

The validity of data in our study is high due to standardized procedures for documenting restrictive practices and BVC. Documentation of restrictive practices in patients' electronic medical records is mandatory, and by law, these must also be reported to the national register for restrictive practices, ensuring completeness and accuracy. Since data is complete and retrospectively collected, the risk of selection bias is minimal. BVC scores are routinely recorded three times daily for each patient by trained staff. Though occasional missing scores may occur when patients are not in the ward or are asleep, we believe these appear randomly both pre‐ and post the relocation. Similarly, if patients are assessed more than three times a day due to changes in their behavior, the highest score was used in our study, which may slightly increase the mean BVC score. Our data collection procedure is robust and has minimal risk of systematic information bias.

A relocation is a complex intervention because it involves not only the physical move from old to new buildings but several other components, such as potential changes in procedures and staff turnover (Skivington et al. 2021). However, in the case of our relocation, patients were admitted to the same wards and remained under the care of the same staff as before the move. There were no changes in legislation or effort. Denmark has been working to reduce restrictive practices since 2014 and has recently set a goal to decrease their use by 40% by 2030 (Sundhedsstyrelsen 2025; Danske‐Regioner n.d.).

The consistency in staffing, the patient population, and ward organization, together with unchanged procedures for restrictive practices and BVC scoring, helps minimize the impact of other variables and strengthens the argument for an association between optimized architecture and reduction of aggressive behavior and restrictive practices. However, given the simultaneous and not easily differentiated changes during relocation, shifts in staff composition or experience cannot be fully excluded as contributing factors.

The immediate decrease in patient aggression and use of restrictive practices post‐relocation may reflect a Hawthorne effect; however, as our study spans 2 years after relocation, and most documented effects rarely persist beyond 6 months, this influence is likely limited (McCambridge et al. 2014).

Our study population is consistent in gender, age, and diagnosis both pre‐ and post the relocation, aligning with similar patient populations from Denmark (Retspsykiatri 2015). Therefore, the results can be generalized to comparable populations within Denmark but also other countries with similar conditions.

### 4.4 Clinical Implication

Several studies (Ulrich et al. 2018; Czernin et al. 2024; Harpøth et al. 2022; Rohe et al. 2017) and reviews (Seppänen et al. 2018; Oostermeijer et al. 2021; Bodryzlova et al. 2024) strengthen the evidence that architecture influences restrictive practices among psychiatric patients. Our findings add a new dimension by suggesting that improved treatment facilities not only reduce the restrictive practices but also lower aggressive behavior. However, our study does not encompass all potential impacts, such as the perspectives of patients and healthcare staff. A recent scoping review underscores that the concept of “healing architecture” lacks a standardized definition and that empirical evidence on its direct influence on clinical and patient outcomes remains limited (Simonsen et al. 2022). However, existing qualitative studies explore how architecture affects patients and staff in psychiatric care. For example, Parkes et al. (2015) interviewed male patients in secure mental health units during relocation from old buildings to new purpose‐built buildings. Patients’ concerns during relocation primarily focus on practical changes, underscoring the need for clear communication and patient involvement throughout the relocation process.

Johnston et al. (2022) also conducted interviews with staff and inpatients across forensic psychiatric wards, emphasizing that increased access to ward spaces improves staff's ability to effectively de‐escalate challenging situations. Integrating qualitative perspectives in future studies may help identify the specific architectural elements that most effectively improve patient outcomes.

A recent meta‐epidemiological study found similar effect estimates in RCTs and observational studies, supporting the validity of our design (Toews et al. 2024). However, larger multicenter studies are needed to confirm the impact of architecture on aggression and restrictive practices in forensic psychiatry.

The increasing amount of literature (Ulrich et al. 2018; Czernin et al. 2024; Harpøth et al. 2022; Rohe et al. 2017; Seppänen et al. 2018; Oostermeijer et al. 2021; Bodryzlova et al. 2024), including our study's results, emphasizes the need for clinicians to consider architectural design as a potential key element in reducing aggressive behavior and, ultimately, restrictive practices. We argue that modernization of psychiatric facilities should be a policy priority to enhance patient care. This involves not only the construction of new hospitals but also the regular updating of existing buildings. Reducing restrictive practices is crucial both for patients and healthcare staff but also for society as the topic raises both legal and ethical considerations (Chieze et al. 2021). This complex issue demands ongoing political attention and sustained financial investments to continue progress.

Our study contributes to understanding how environmental changes may influence patient outcomes and may be relevant for design and renovation of psychiatric hospitals. While specific architectural features could not be isolated, our comparison of the old and new settings (Table 1) may guide targeted improvements. We emphasize the need for ongoing updates to psychiatric facilities to meet evolving healthcare standards. Maintenance of psychiatric buildings and the interior design is crucial to preserving a safe, secure, and supportive environment for both patients and staff. Without continuous upgrades, the facilities become outdated, potentially affecting the quality of care. Consequently, investing in design innovation and long‐term upkeep is essential when providing and improving psychiatric healthcare.

## 5 Conclusion

This retrospective, observational, and longitudinal study suggests a significant reduction in overall aggression and prescription of restrictive practices following the relocation from old hospital buildings to newly purpose‐built facilities in a population of forensic psychiatric inpatients. This decline was observed across multiple categories of aggression levels and restrictive interventions, as evidenced by both summative data and underlying time trends. Our findings indicate that optimizing treatment facilities may contribute to reduced patient aggression and fewer restrictive practices. While the findings suggest that improved facilities contribute to these outcomes, further research is needed to identify the specific architectural factors involved.

## Author Contributions

L.K. and L.U.S. designed the study protocol with input from A.J.H., A.H.C., M.D.T., C.J., and H.K. A.H.C., L.K., and L.U.S. performed data analysis with input from A.J.H., M.D.T., C.J., and H.K. L.K. drafted the manuscript, and all authors revised and approved the final manuscript.

## Ethics Statement

Central Denmark Region has approved access to the medical records according to The Health Act, § 46, section 2; reference 1‐45‐70‐36‐24 and section 5; reference 1‐16‐5‐72‐719‐23.

## Clinical Trial Registration

The study is registered in the internal list of research projects in the Central Denmark Region; reference 1‐16‐02‐137‐24.

The protocol is available at the Open Science Forum; reference https://doi.org/10.17605/OSF.IO/JHDP8.

## Artificial intelligence statement

During the preparation of this work the authors used ChatGPT for language revision. After using this tool/service, the authors reviewed and edited the content as needed and take full responsibility for the content of the publication.

## Patient Consent Statement

No individual patients have been contacted.

## Conflicts of Interest

The authors declare no conflicts of interest.

## Peer Review

The peer review history for this article is available at https://publons.com/publon/10.1002/brb3.70760

## Data Availability

Data are not available.
